# Use of End-of-Life Care Pathways in Hospitalized Stroke Patients: A Retrospective Study of the AMBER Care and Dying Adults in the Last Days of Life Approaches

**DOI:** 10.3390/healthcare13161979

**Published:** 2025-08-12

**Authors:** Dariusz Kotlęga

**Affiliations:** 1 Department of Pharmacology and Toxicology, University of Zielona Gora, 65-046 Zielona Gora, Poland; dkotlega@uz.zgora.pl; 2 Northampton General Hospital, Northampton NN1 5BD, UK

**Keywords:** stroke, end-of-life care, palliative care, Dying Adults in the Last Days of Life, AMBER Care Bundle, quality of care, neurology

## Abstract

**Background**: Stroke-related deaths often follow rapid deterioration, making end-of-life (EOL) care decisions particularly challenging in acute settings. Although national guidelines support structured approaches to end-of-life care, there is limited evidence of how these pathways are applied in routine stroke practice. **Objective**: To evaluate the use of structured end-of-life care pathways, including the AMBER Care Bundle and Dying Adults in the Last Days of Life (DALDL), in stroke patients who died during admission at a general hospital stroke center. **Methods**: This retrospective, single-center cohort study included 123 patients with confirmed stroke (73.2% ischemic, 26.8% hemorrhagic) who died in hospital during 2023. Clinical characteristics, the timing of care pathway decisions, palliative care involvement, withdrawing of medical procedures, and outcomes were analyzed. Descriptive statistics, Mann–Whitney U tests, Spearman correlations, chi-square tests, and a multivariate regression model were performed. **Results**: Of 123 patients, 101 (82.1%) entered the DALDL pathway a median of 14.8 days after admission, with a subsequent median survival of 2.9 days. Anticipatory medications were prescribed in 100% of DALDL patients versus 0% of non-DALDL. Do Not Attempt Cardiopulmonary Resuscitation orders were documented in 99%, and 67.3% received specialist palliative care input. Nasogastric tube insertion correlated with a higher National Institutes of Health Stroke Scale (NIHSS) and higher rate of infections. **Conclusions**: Most patients had access to structured EOL care, but variability in timing and interventions highlights the need for earlier palliative engagement and consistent implementation of pathways to improve the quality of EOL care in stroke patients. We detected areas that could be improved, such as access to a palliative care team and the anticipatory medication use in dying stroke patients.

## 1. Introduction

Stroke is the second leading cause of death, and the third leading cause of death and disability combined worldwide. The global incidence rate is estimated at 142–151 per 100,000 population. Ischemic stroke (IS) accounts for 65.3–74.9% of cases, intracerebral hemorrhage (ICH) for 28.8%, and subarachnoid hemorrhage (SAH) for 5.8%. Stroke-related deaths increased by 44% globally between 1990 and 2021, and are projected to continue rising in the coming years [1,2]. The 30-day mortality rate in the UK is 11.9% for ischemic stroke and 30.5% for intracerebral hemorrhage [3]. In-hospital fatality rates range from 2.1% for all strokes to 17.5% for hemorrhagic strokes in high-income countries [4,5]. According to other sources, the overall in-hospital mortality rate for all strokes is approximately 9% [6]. Stroke accounts for approximately 11% of all deaths in England and Wales [7].

Approximately half of all stroke-related deaths occur in hospitals, with 35% occurring in care homes and 15% at home or in other facilities. Given the significant number of fatalities, access to appropriate end-of-life care pathways is essential to ensure a comfortable environment and to treat patients with dignity during their final days. Access to palliative care and structured end-of-life pathways for stroke patients is vital for effective symptom control and for enabling person- and family-centered care. The goals of palliative services include optimizing quality of life by reducing suffering and addressing social, emotional, physical, cognitive, and spiritual needs. Current recommendations for stroke care emphasize the importance of patient- and family-centered approaches. Clinicians should discuss the prognosis with each patient and their family, drawing on both the relevant literature and their own clinical experience. In situations of uncertainty, seeking a second opinion should be considered, particularly when making decisions regarding the initiation of end-of-life care or the limitation of treatment. An important component of in-hospital end-of-life care is the implementation of Do Not Attempt Cardiopulmonary Resuscitation (DNACPR) orders and Treatment Escalation Plans (TEPs). These outline decisions regarding various aspects of care, including cardiopulmonary resuscitation, nutrition, medications (such as antibiotics and inotropes), mechanical ventilation, and admission to intensive care units [8]. In addition to prognostic uncertainty, barriers to providing appropriate palliative care for stroke patients may include limited access to standardized protocols and a shortage of palliative care specialists. The overarching aim of palliative care is to improve the quality of life for patients and their families [9].

Stroke may result in a range of complications, including pain, distress, cognitive impairment, depression, confusion, agitation, and difficulties with hydration and nutrition. Addressing these issues through a holistic approach is essential to delivering high-quality end-of-life (EOL) care for stroke patients [10]. The EOL care pathway is intended for patients with an uncertain prognosis despite treatment, and provides care, support, planning, and preparation in the final weeks or months (occasionally years) of life. EOL care includes individuals with progressive, advanced, incurable conditions; those with general frailty and comorbidities that increase the risk of dying within the next 12 months; individuals with existing conditions who are at risk of sudden acute deterioration; and patients with life-threatening acute conditions resulting from catastrophic events. The identification of such patients can be supported by structured tools, including the Gold Standards Framework, the AMBER Care Bundle, and the Supportive and Palliative Care Indicators Tool (SPICT) [11].

The AMBER Care Bundle and the Dying Person’s Care Plan are palliative care pathways that may be applied to stroke patients to support dignified and compassionate care. The AMBER Care Bundle, introduced in 2010, is intended for patients whose recovery is uncertain despite active treatment [12]. Typically, these patients have a life expectancy of weeks to several months. AMBER stands for Assessment, Management, Best practice, Engagement, and Recovery uncertain. This approach aims to enhance dignity and respect, improve communication and shared decision-making, reduce anxiety, facilitate home deaths where preferred, and equip staff with the knowledge, confidence, and skills needed to deliver effective care. It may also help reduce emergency readmission rates [13].

There are specific recommendations for the care of dying adults in their final two to three days of life. Once a person is recognized as approaching the end of life, clinicians must make decisions about individualized care. These decisions should take into account the patient’s personal goals, wishes, and preferences for symptom management, including pharmacological interventions and the discontinuation of medications that are no longer necessary for symptom control. The UK National Institute for Health and Care Excellence (NICE) provides guidance for the care of Dying Adults in the Last Days of Life (DALDL), which can be implemented in clinical practice. Symptom control is often achieved through the use of anticipatory medications, which are prescribed to manage common symptoms such as pain, breathlessness, nausea and vomiting, anxiety, delirium, agitation, and noisy respiratory secretions. Decisions regarding artificial hydration and nutrition should consider the expressed preferences of the patient and their family, the patient’s level of consciousness, swallowing ability, degree of thirst, and the risk of pulmonary oedema. Commonly used anticipatory medications include morphine, haloperidol, midazolam, hyoscine, and glycopyrronium [14]. Distressing symptoms frequently observed in hospitalized stroke patients include tiredness (83%), drowsiness (58%), anorexia (57%), delirium (50%), pain (36%), breathlessness (26%), respiratory secretions (26%), anxiety (24%), constipation (13%), nausea (10%), and depression (8%) [15]. Other studies highlight additional challenges managed by palliative care teams in stroke patients, including dyspnea (30%); pain (25–30%, typically central post-stroke pain, hemiplegic shoulder pain, and pain due to spasticity); xerostomia (20%); constipation (20%); sadness (35–50%); anxiety (25%); and fatigue (50%) [16].

The most recent recommendations for stroke end-of-life care from the American Heart Association (AHA) underscore the importance of a comprehensive assessment of multidimensional distress. This includes physical, emotional, psychosocial, spiritual, and existential dimensions [17]. End-of-life care is a critical component of stroke management, particularly for patients with a severe neurological impairment or significant comorbidities. Currently, there are limited data describing real-world patterns of end-of-life care, DALDL decision-making, symptom management, and the transition to palliative care. The challenges which are related to the last period of life in stroke include the inconsistent use of EOL pathways, delayed recognition of dying patients, delayed decisions regarding feeding, or limited integration of palliative care in stroke settings. There are also limited real-life data regarding these aspects of care in stroke wards, recommendations for timing, intervention use or discontinuation, as well as predictors of end-of-life decisions.

The aim of this study was to analyze the care provided to stroke patients during their final days of life, with the intention of discussion and improving services for dying patients. This was especially important due to the limited real-world data on structured EOL pathways in stroke. In this retrospective single-center cohort study on the acute stroke ward, the author aimed at quantifying the timing of initiation of two structured end-of-life pathways, the AMBER Care Bundle and the DALDL guidance, among adult patients aged eighteen years and older with confirmed ischemic or hemorrhagic stroke who died during the same admission. The goal was also to describe setting-specific interventions and the support received, including specialist palliative care nurse involvement, DNACPR orders, withdrawal of life-prolonging treatments, nasogastric feeding, anticipatory medication prescribing, and associations between certain aspects of EOL care and clinical variables.

## 2. Material and Methods

### 2.1. Study Design and Setting

This retrospective, single-center cohort study was conducted on the acute stroke ward of a National Health Service (NHS) general hospital in the United Kingdom. A total of 123 patients were included in the study: 112 patients (91.1%) were admitted directly to the stroke ward, while 11 patients (8.9%) were admitted to other wards, but were also under the care of the stroke team.

The study was conducted in accordance with the Declaration of Helsinki and the UK Data Protection Act (2018). It was registered with the local Clinical Audit and Improvement department number stroke/SE/2025-26/01. As a retrospective analysis, patient consent was waived. The NHS Health Research Authority confirmed that review by an NHS Research Ethics Committee was not required.

### 2.2. Data Collection

**Figure 1:**
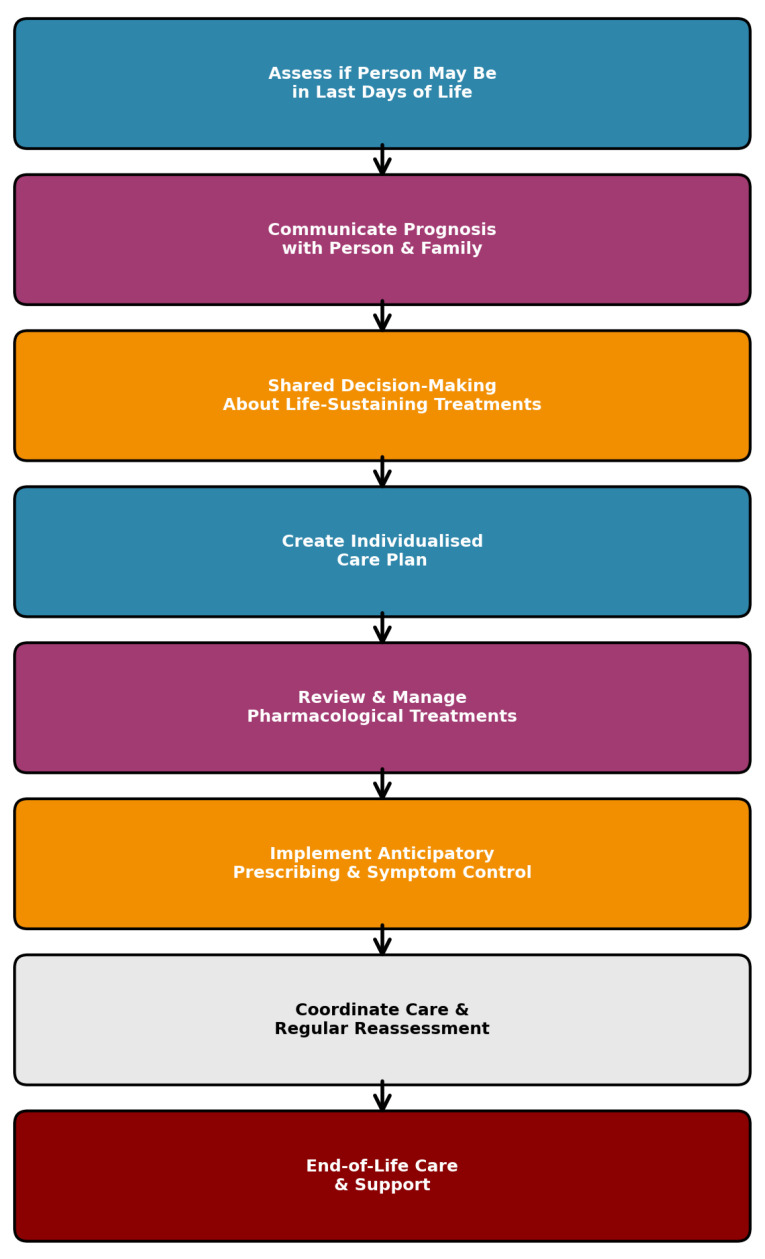
The flowchart of Dying Adult in the Last Days of Life pathway.

Data were collected over a 12-month period, from 1 January 2023 to 31 December 2023. The study included all patients aged ≥18 years who were admitted with a radiologically and clinically confirmed diagnosis of acute stroke, either an ischemic stroke or intracerebral hemorrhage, who died during the same hospital admission. Demographic and clinical data were obtained from hospital records. The following information was collected: age, sex, baseline functional status using the modified Rankin Scale (mRS), stroke subtype (ischemic or hemorrhagic), stroke severity on admission using the National Institutes of Health Stroke Scale (NIHSS), consciousness level, major comorbidities, including atrial fibrillation, malignancy, dementia, chronic heart failure, and chronic kidney disease or previous stroke/TIA (Transient Ischemic Attack). Additional data included end-of-life decision-making, documentation of Do Not Attempt Cardiopulmonary Resuscitation (DNACPR) orders, withdrawal of life-prolonging interventions, specialist palliative care nurse consultations, nasogastric (NG) tube insertion, use of antibiotics, and detection of infection [18,19]. As part of the assessment, information regarding the implementation of end-of-life care pathways, specifically the AMBER Care Bundle and the DALDL pathway, was also collected. The designation of DNACPR, the AMBER Care Bundle, and the DALDL pathway was determined from the multidisciplinary team notes and formal pathway documentation in the patient’s electronic medical records. The flowchart describing the Dying Adult in their Last of Days of Life was presented in Figure 1.

Every end-of-life decision according to the hospital forms and guidelines must be approved by a senior physician (consultant), and these decisions are documented in the patient’s medical records. DNACPR decisions were made during admission by the admitting physician or during the later stages of hospital stay by ward-based physicians. All forms were signed and documented as part of the patient’s medical records. No patient gave informed consent for the DALDL pathway; in all cases, it was documented that they lacked capacity. All decisions were discussed and agreed with the next of kin, which was documented in the dedicated form. Although these are medical decisions, the good practice is to discuss and confirm these decisions with the next of kin.

The cause of death was determined based on the death certificate and clinical judgement after analyzing the course of the hospital stay. The categories, such as stroke/ICH, infection, and cardiac, indicated the predominant cause of death. The category of “other” indicated the cause of death, which was not listed in the main categories, while “complex” was used when multifactorial causes were identified.

There were 6 patients excluded from the study, 5 due to missing electronic records and 1 due to the diagnosis of post-traumatic subdural hematoma. Other clinical data were complete.

### 2.3. Statistical Methods Summary

Continuous variables were inspected for normality with the Shapiro–Wilk test and summarized as the mean ± standard deviation (SD) or median (interquartile range, IQR) as appropriate. Categorical variables were presented as counts and percentages. Associations between timing and clinical/laboratory variables were assessed using Spearman correlation for continuous predictors, the Mann–Whitney U test for binary predictors, and the Kruskal–Wallis test for categorical predictors with more than two groups. Chi-square tests and a multivariate regression model was used. All analyses were performed using Python 3.11 (pandas 1.5, SciPy 1.11, and seaborn 0.12). A significance threshold of *p* < 0.05 was used. Power calculations of the sample size indicated that this study was appropriate for descriptive conclusions, but the margins of error are 9% for key proportions. The calculated power analysis for the timing of care pathways was >99%, for AMBER Care rate comparison was 58%, and for DALDL rate comparison was 35%. The presented significant findings were confirmed by Bonferroni correction with corrected significance level α = 0.000714.

## 3. Results

### 3.1. Overall Cohort Description and Survival

The cohort comprised 123 patients who died during their hospital stay, with a mean age of 79.4 years (range: 40 to 99 years; SD ± 10.4). Of these, 73% (n = 90) had ischemic strokes and 27% (n = 33) had intracerebral hemorrhage (ICH). The mean National Institutes of Health Stroke Scale (NIHSS) score on admission was 13.4 ± 7.6. The characteristics of the entire cohort are presented in Table 1.

**Figure 2:**
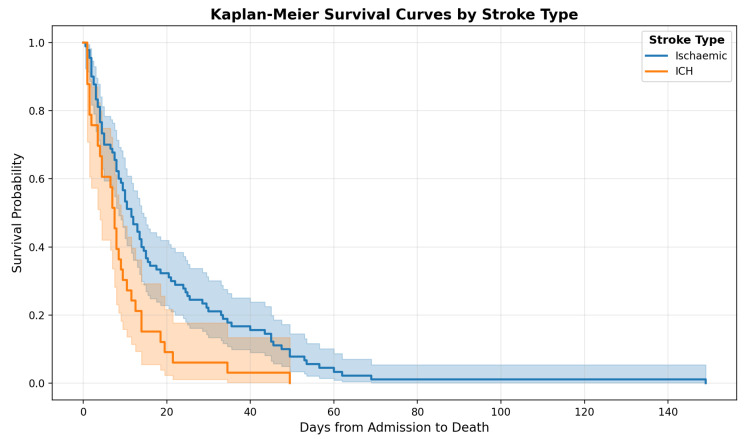
Survival curve in ischemic and hemorrhagic stroke patients.

The Kaplan–Meier survival curves (Figure 2) illustrate the probability of survival from admission for patients with ischemic stroke compared to those with intracerebral hemorrhage (ICH). Patients with ischemic stroke (n = 90) had a median survival of 11.5 days, whereas patients with ICH (n = 33) had a shorter median survival of 7.5 days. This difference was statistically significant (Mann–Whitney U test, *p* = 0.008). When analyzing only patients identified under the Dying Adults in the Last Days of Life (DALDL) pathway, a similar trend was observed; however, the difference did not reach statistical significance (*p* = 0.053).

### 3.2. AMBER Care and Dying Adults in the Last Days of Life Pathways

**Figure 3:**
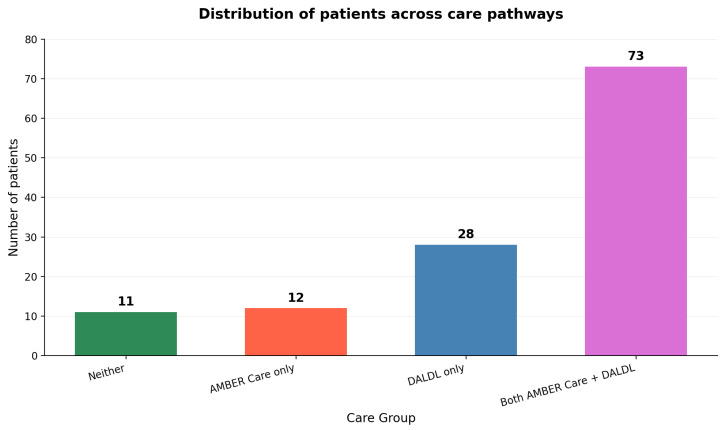
Distribution of AMBER Care and DALDL pathways.

Among all patients, 10 (8.1%) were on neither the AMBER Care Bundle nor the Dying Adults in the Last Days of Life (DALDL) pathway. Twelve patients (9.8%) were on AMBER Care only, 28 patients (22.8%) were on DALDL only, and 73 patients (59.3%) were on both AMBER Care and DALDL (Figure 3). Overall, 85 out of 123 patients (69.1%) received AMBER Care.

A total of 101 patients were identified as meeting the criteria for the Dying Adults in the Last Days of Life (DALDL) pathway. The median age was 81 years, and 41 patients (40.6%) were male. This group accounted for 82.1% of all in-hospital deaths. Among the DALDL patients, 74 (73.3%) were diagnosed with ischemic stroke, while 27 (26.7%) had intracerebral hemorrhage (ICH). The mean NIHSS score on admission was 13.5 ± 7.3. The characteristics of this patients’ group are presented in Table 2.

For patients on the DALDL pathway, AMBER Care was initiated on average 9.6 days after admission (median: 5 days; *n* = 73). In contrast, for patients not on the DALDL pathway, AMBER Care was initiated significantly earlier, with a median of 0.5 days (mean: 0.83 days; *n* = 3), a difference that was statistically significant (Mann–Whitney U test, *p* = 0.036). The median time from AMBER Care initiation to death was 7.2 days.

The mean time from admission to DALDL initiation was 14.8 days (SD ± 20.7), and the mean duration from DALDL initiation to death was 2.9 days (SD ± 2.9). The overall mean length of stay for all patients was 16.6 days (SD ± 19.9; range: 1 to 149 days). After adjusting for outliers, the mean time from admission to death was 12 days (SD ± 11).

### 3.3. Comparison of DALDL and Non-DALDL Patients

Analysis of the causes of death revealed no significant differences between patients classified within the DALDL group and those in the non-DALDL group. The distribution of causes of death is presented in Table 3 and the statistical analysis confirmed no significant difference between the groups (Chi-square test, *p* = 0.28).

A comparison of selected parameters and comorbidities between DALDL and non-DALDL patients revealed borderline significance in length of stay (*p* = 0.056) and a statistically significant difference in sex distribution. In the DALDL group (n = 101), the mean length of stay was 17.8 days (SD ± 21.1), compared to 11.1 days (SD ± 12.7) in the non-DALDL group (n = 22). There was a female predominance in the DALDL group (n = 60, 59.4%), whereas male patients were significantly more frequent in the non-DALDL group (n = 17, 77.3%), with a Chi-square test *p* value of 0.039. After adjusting for confounding variables in a multivariable regression model, the difference in sex distribution remained significant (*p* = 0.0026), which was presented in Table 4.

### 3.4. Medical Interventions in DALDL Patients

**Figure 4:**
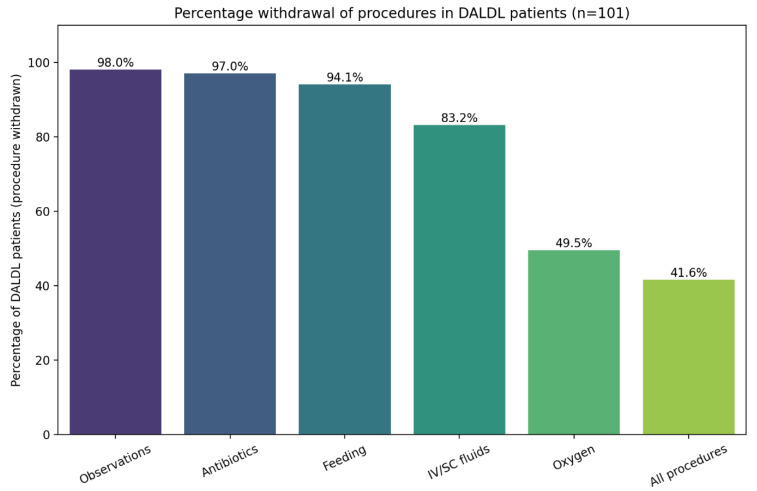
Distribution of procedure withdrawal in DALDL patients. Legend: IV—intravenous, SC—subcutaneous.

Figure 4 presents the percentage of specific procedures withdrawn in DALDL patients. No clinical parameters showed a significant association with this outcome.

### 3.5. Nasogastric Tube Insertion

A nasogastric (NG) tube was inserted in 61 patients (49.6% of the total cohort) and in 52 patients (51.5%) within the DALDL group. The median of NG tube insertion in DALDL patients was 2 days, while from this intervention to death, the median time was 22.8 days. There was no significant difference in the frequency of NG tube insertion between the DALDL group and other patients (*p* = 0.48).

Clinical predictors of NG tube insertion among DALDL patients included the admission NIHSS score, the interval from initiation of AMBER Care to DALDL, and the time from DALDL initiation to death (Table 5). Furthermore, NG tube insertion was significantly more likely in DALDL patients with infection who received antibiotics (87% vs. 65%; *p* < 0.05; odds ratio [OR] 3.14).

Nasogastric (NG) tube insertion occurred on average 3.37 ± 4.9 days after admission for the full cohort, with a mean duration from NG tube insertion to death of 18.84 ± 23.31 days. For DALDL patients specifically, these timings were 3.44 ± 4.95 days and 19.08 ± 24.66 days, respectively. 

Within the DALDL group, the time from admission to NG tube insertion showed a strong negative correlation with NIHSS scores (Spearman’s *r* = −0.54, *p* < 0.001), and a positive correlation with Glasgow Coma Scale (GCS) scores (*r* = 0.33, *p* = 0.018). It was also significantly associated with the presence of dysphasia (*p* = 0.03) and atrial fibrillation (*p* = 0.03). Other variables, including comorbidities, age, and laboratory values, did not demonstrate significant associations.

The time from NG tube insertion to death was positively correlated with the total length of stay (*r* = 0.815, *p* < 0.001), time from admission to initiation of the DALDL pathway (*r* = 0.697, *p* < 0.001), time from initiation of AMBER Care to DALDL (*r* = 0.599, *p* < 0.001), involvement of a palliative care nurse (*r* = 0.356, *p* = 0.0095), and time from DALDL initiation to death (*r* = 0.352, *p* = 0.01). A negative correlation was observed with the stroke subtype, specifically hemorrhagic stroke (*r* = −0.278, *p* < 0.05).

### 3.6. Palliative Care

Palliative care nurses were involved significantly more frequently in the care of patients within the DALDL group (n = 68, 67.3%) compared to the non-DALDL group (n = 0), with this difference reaching statistical significance (*p* < 0.001). There were no significant differences between the groups regarding stroke subtype, age, NIHSS and GCS scores, infections, antibiotic use, or comorbidities. Overall, 68 patients (55.3%) had access to the palliative care team. Among patients on the AMBER Care pathway, 65.9% (n = 56) received palliative care nurse involvement, compared to 36% (n = 12) of patients not on the AMBER Care pathway; this difference was statistically significant (Fisher’s exact test, *p* = 0.0016). Additionally, 52.2% of patients with ischemic stroke and 63.6% of those with intracerebral hemorrhage had access to palliative care, although this difference was not statistically significant (*p* = 0.35).

Anticipatory medications were prescribed in all of the DALDL patients (n = 101), in 85.9% of AMBER Care patients (n = 73) and in 81.5% of all patients (n = 101). Among 12 patients who were on AMBER Care only, none of them received anticipatory medications.

## 4. Discussion

We evaluated a cohort of 123 patients who died during the hospital admission in which their stroke was diagnosed. The median survival was significantly shorter for those with intracerebral hemorrhage (7.5 days) than for those with ischemic stroke (11.5 days), reflecting the generally poorer prognosis associated with ICH.

The mean time from admission to entry onto the DALDL pathway was 14.8 days (SD ± 20.7), and the mean interval from DALDL initiation to death was 2.9 days (SD ± 2.9). The overall mean length of stay was 16.6 days (SD ± 19.9), which decreased to 12 days after adjustment for outliers. In another stroke cohort, the mean time from admission to death was 7 days (range 1–30), and 2.6 days from therapy restriction to death, compared with 2.9 days in our study. As observed in the current study, the mean NIHSS score on admission (13.4) was lower than reported by other authors (mean 19), which may partly explain why we observed a later decision to restrict therapy. Patients in the comparator cohort were diagnosed both with ischemic and ICH in Germany, and were evaluated retrospectively to assess the end-of-life decisions and care on the stroke ward. In a cohort of stroke patients in Norway, the median time from withdrawing life-sustaining treatment to death was 4 days, from admission to death was 9.8 days, and the NIHSS score was 22 points [20]. Nevertheless, delayed recognition and decision-making underline the need for earlier identification of patients with poor prognoses, given the current lack of clear, standardized guidelines. Do Not Attempt Cardiopulmonary Resuscitation (DNACPR) orders were documented in 99% of our cohort, compared to 86.3% in the literature [4].

As much as 101 (82.1%) persons were entered onto the DALDL pathway and analyzed in greater detail. As presented in Figure 3, 85 patients (69.1%) received the AMBER Care Bundle, and 113 patients (91.3%) were transitioned to some form of end-of-life care. Non-DALDL patients experienced a more gradual decline and longer hospital stays, consistent with less severe disease. Only 8.1% of patients received no formal EOL pathway, 9.8% were on AMBER Care only, 22.8% on DALDL only, and 59.3% on both pathways. Thus, the vast majority had access to structured EOL support during their admission. The relatively short interval between DALDL initiation and death underscores the accuracy with which patients were identified as their final days. However, the wide variability in timing and choice of interventions suggests room for greater standardization and earlier engagement of specialist palliative care services. On the other hand, such decisions are complex and eventually it might still be difficult to implement highly precise standardization due to interpersonal differences in the process of dying, individual and carer’s wishes, and certain unpredictability in such clinical circumstances.

All DALDL patients received anticipatory medications for symptom control, whereas those not formally on the pathway did not. Overall, 81.5% of the cohort received such medications. Of note is that among 12 patients who were on AMBER Care only, none of them received anticipatory medications. High rates of DNACPR orders and anticipatory prescribing in DALDL patients indicate adherence to best practice, although broader access, particularly for patients on AMBER Care only, might further improve comfort and dignity. Anticipatory medications are crucial for alleviating pain, dyspnea, dry mouth, agitation, and respiratory secretions, the most distressing symptoms encountered in this population [17,21]. Raising medical professionals’ awareness of the importance of broader anticipatory medication use could extend these interventions beyond the DALDL pathway. As a result, more dying stroke patients would benefit from such medications.

Among DALDL patients, 67.3% of them had access to a palliative care nurse. Lack of palliative care in some of the patients partly reflects the rapid progression to death in some cases shortly after the formal initiation of the DALDL pathway. Overall, just 55.3% of all patients accessed the specialist palliative care team. Of those who did, 72.3% had previously been started on the AMBER Care Bundle. Conversely, only 65.9% of all AMBER Care patients went on to receive palliative care input. Combining both end-of-life pathways (AMBER Care and/or DALDL), 68 patients (60.2%) had palliative care nurse involvement. In a UK survey of stroke clinicians, 44% reported that every patient transitioning to end-of-life care was referred to a specialist palliative care team, and in a separate survey, all respondents working on stroke wards agreed that palliative care is an essential component of stroke management [22,23]. Our cohort demonstrated higher referral rates to specialist palliative care than other series, in which only 38% of in-hospital stroke deaths received such services [6]. Those authors also observed that patients with ICH were more likely to access palliative care, a finding not replicated in our study.

Improving access to specialist palliative care is likely to enhance the quality of end-of-life care for stroke patients. Early identification of patients suitable for the AMBER Care Bundle, coupled with prompt involvement of palliative care services, should be a key priority. The AMBER Care pathway targets patients with an anticipated life expectancy of months (typically up to a year), a group that encompasses a large proportion of those with severe strokes. According to this analysis, being placed on the AMBER pathway more than doubles the likelihood of receiving specialist palliative care input. Once patients are flagged as AMBER, clinicians appear more proactive in involving palliative teams, whereas those never flagged, even if they have equally disabling strokes, are much less likely to receive this support. In this study, it was observed that the AMBER Care Bundle was initiated on average 9.6 days after admission (median 5 days), and the median time from this decision to death was 7.2 days. There are no similar cohorts available to compare, but in the analysis of acute medical wards in a UK hospital, the median time was 10 and 9 days, respectively. The comparator cohort consisted of cancer (25%), non-cancer (31%), and multimorbidity (44%) as admission diagnoses. In the other cohort, patients were more likely to be supported by AMBER Care when their illness trajectory was predictable and gradual, rather than rapid or unpredictable [24]. The timing of NG tube insertion accounted for 2 days, which seems to be too long, as it is recommended to establish NG tube decisions in the first 24 h [25].

There was also an analysis of the withdrawal of life-prolonging interventions in DALDL patients (Figure 4). Observations were discontinued in 98% of cases, antibiotics in 97%, enteral feeding in 94.1%, intravenous fluids in 83.2%, and oxygen in 49.5%, while all interventions were stopped in 41.6% of patients. The variability in which interventions are withdrawn, particularly oxygen, which may both prolong life and enhance comfort, highlights the lack of clear, procedure-specific guidance. Finally, it was noted that there was a predominance of female patients in the subgroup with complete intervention withdrawal, a pattern that warrants further investigation to understand potential gender-based differences in end-of-life decision-making. On the other hand, this finding could have been caused by the small sample size. In a similar cohort, observations, antibiotics, and feeding decisions were implemented to a similar extent (95%, 86%, and 98%, respectively), but contrary to our cohort, all patients were receiving IV fluids adapted to palliative care needs [4]. This significant difference might reflect local practice, which clearly highlights the field for further discussion and recommendations.

As much as 51.5% of DALDL patients had an NG tube inserted. Other authors have reported that 94.9% of palliative stroke patients ultimately received a percutaneous endoscopic gastrostomy (PEG), presumably following NG tube placement [6]. On average, NG tube insertion is provided in 8.8% of all stroke patients, but the studied cohort included a selected group of patients, with more severe ailments [26]. In the studied cohort, higher odds of NG tube insertion were positively correlated with the admission NIHSS score, time from AMBER Care initiation to DALDL, time from DALDL initiation to death, and the presence of infection with corresponding antibiotic use (Table 5). Stroke severity clearly drove both the likelihood and the rapidity of NG tube insertion: higher NIHSS scores increased insertion probability, while lower GCS scores at admission were associated with shorter times to insertion. Although dysphasia and atrial fibrillation correlated with delayed insertion, these relationships do not imply causation and may instead reflect underlying clinical complexity. Both decisions regarding the insertion and removal of an NG tube might be challenging. On the one hand, NG tube feeding is an important medical procedure that improves outcomes in stroke patients, but on the other hand, it is one of the interventions that might need to be withdrawn in the EOL pathway. The complexity of such decisions and need for the next-of-kin’s involvement were discussed by other authors [27,28].

This study offers a detailed appraisal of end-of-life care practices on a UK stroke ward over one year. High rates of anticipatory prescribing, DNACPR documentation, and use of the AMBER Care and DALDL pathways indicate an encouraging integration of structured palliative approaches into stroke management. Nonetheless, considerable variability in timing, suboptimal anticipatory medication coverage, limited early palliative care involvement, and inconsistent withdrawal of life-prolonging interventions highlight the need for further standardization and clearer guidance to support timely, patient-centered decision-making. Moreover, terminology remains inconsistent: in a UK survey, medical staff, patients, and families often equated “palliative care” with the “last days of life,” potentially leading to confusion and late referrals [29].

The main limitations of this study are sample size, retrospective design, and reliance on routinely recorded electronic healthcare records. Such a design might mean that such variables as clinical decisions or symptom burden assessments may have been inconsistently documented or subject to misclassification. The non-DALDL group consisted of only 22 patients and statistical comparisons between pathway groups are underpowered. The power calculations indicate that the presented results may be useful for descriptive conclusions only, while more detailed analyses must be interpreted with caution. This study was confined to a single stroke ward, and the findings may not generalize to other settings with different patient demographics, staffing models, or end-of-life care protocols. Another limitation is that the patient- or family-reported experiences of care and wishes were not captured, but these factors influence the clinical decision-making.

## 5. Conclusions

Early identification of individuals approaching the end of life can be facilitated by enhanced prognostic tools, multidisciplinary collaboration, and proactive engagement by medical staff. Further implementation of prognostication tools might be helpful in the initiation of EOL pathways [30]. Such measures have the potential to improve the quality of care, optimize patient comfort, and enhance the experience of families. Integrating early access to specialist palliative care teams within stroke units should be a priority in quality-improvement initiatives. Transitioning from active treatment to comfort-focused care in stroke patients is inherently complex and requires personalized decision-making. Comprehensive palliative care which emphasizes symptom control and holistic support remains a cornerstone of excellence in stroke management.

## Figures and Tables

**Table 1:** Characteristics of all patients (n = 123).

Comorbidity	n	%
Hypertension	110	89.4
Diabetes	30	24.4
Atrial fibrillation	50	40.7
Heart failure	35	28.5
Ischemic heart disease	26	21.1
Previous stroke / TIA	22	17.7
Dementia	31	25.2
Chronic kidney disease	26	21.1
Cancer	28	22.6
Infection	90	72.6
Antibiotics	90	72.6
Stroke type: ischemic	90	73.2
Stroke type: hemorrhagic	33	26.8

**Table 2:** Characteristics of DALDL patients.

Variable	n	%
Hypertension	90	89.10891
Diabetes	24	23.76238
Atrial fibrillation	40	39.60396
Heart failure	26	25.74257
Ischemic heart disease	21	20.79208
Previous stroke/TIA	17	16.83168
Dementia	26	25.74257
Cancer	24	23.76238
Chronic kidney disease	19	18.81188
Infection	77	76.23762
Antibiotics	77	76.24
Stroke type: ischemic	74	73.3
Stroke type: hemorrhagic	27	26.7
Anticipatory medication	101	100
AMBER Care	73	72.3
Palliative care nurse	68	67.3
DNACPR	100	99
NG tube	52	51.5
Cause of death: stroke/ICH	27	26.7
Cause of death: infection	15	14.9
Cause of death: cardiac	2	2
Cause of death: other	7	6.9
Cause of death: complex	50	49.5

**Table 3:** Distribution of causes of death in DALDL and non-DALDL groups.

Variable	DALDL	Non-DALDL	*p*-Value
Cause of death: stroke/ICH	27 (26.7%)	6 (27.3%)	NS
Cause of death: infection	15 (14.9%)	6 (27.3%)	NS
Cause of death: cardiac	2 (2%)	1 (4.5%)	NS
Cause of death: other	7 (6.9%)	3 (13.6%)	NS
Cause of death: complex	50 (49.5%)	6 (27.3%)	NS
Length of stay	17.8 days (SD ± 21.1)	11.1 days (SD ± 12.7)	*p* = 0.056
Female sex	60 (59.4%)	17 (77.3%)	*p* < 0.005
Palliative care	68 (67.3%)	0	*p* < 0.001

**Table 4:** Multivariate analysis of confounding factors on comparison between DALDL and non-DALDL groups.

Variable	Odds Ratio (95% CI)	*p*-Value	Significance
DALDL status	46.01 (3.60–587.87)	0.003	*
Age	1.10 (1.00–1.21)	0.045	*
Premorbid mRS	1.56 (0.70–3.47)	0.273	NS
Type of stroke (ischemic/ICH)	1.84 (0.37–9.11)	0.454	NS
NIHSS admission	1.12 (0.96–1.30)	0.141	NS
GCS	1.05 (0.69–1.60)	0.814	NS
Dementia	1.98 (0.23–17.35)	0.538	NS
Hypertension	0.88 (0.08–9.97)	0.921	NS
Diabetes	1.37 (0.23–8.06)	0.724	NS
Atrial fibrillation	1.09 (0.21–5.68)	0.914	NS
Congestive heart failure	0.08 (0.01–0.65)	0.018	*
Ischemic heart disease	1.99 (0.30–13.11)	0.474	NS
Previous stroke/TIA	1.89 (0.18–19.35)	0.593	NS
Cancer	1.93 (0.34–10.81)	0.456	NS
Chronic kidney disease	0.67 (0.08–5.81)	0.715	NS

* statistically significant *p*-value; NS statistically non significant.

**Table 5:** The factors affecting NG tube insertion in DALDL patients.

Variable	Non-NG Tube (Mean, SD)	NG Tube (Mean, SD)	*p*-Value
NIHSS admission	11.1 ± 7.29 (n = 49)	15.67 ± 6.72 (n = 52)	0.0015
AMBER to DALDL time (days)	5.97 ± 9.94 (n = 33)	12,57 ± 13.82 (n = 40)	0.0207
DALDL to death time (days)	2.26 ± 2.46 (n = 49)	3.61 ± 3.26 (n = 52)	0.0206
Cause of death: complex/multifactorial	28.6% (n = 14)	69.2% (n = 36)	0.00006
Cause of death: other	14.3% (n = 7)	0% (n = 0)	0.005
Cause of death: stroke/ICH	36.7% (n = 18)	17.3% (n = 9)	0.042

## Data Availability

The raw data supporting the conclusions of this article will be made available by the authors on request.
